# Pain Modulation and Central Sensitization in Painful Diabetic Peripheral Neuropathy: Updated Narrative Review and Future Directions

**DOI:** 10.1111/1753-0407.70223

**Published:** 2026-04-03

**Authors:** Di Ye, Timothy J. Fairchild, Lechi Vo, Peter D. Drummond

**Affiliations:** ^1^ School of Psychology and Centre for Healthy Ageing, College of Health and Education, Murdoch University Murdoch WA Australia; ^2^ School of Allied Health and Centre for Healthy Ageing, College of Health and Education, Murdoch University Murdoch WA Australia

**Keywords:** central sensitization, diabetes, hyperglycemia, metabolic syndrome

## Abstract

Painful diabetic peripheral neuropathy (DPN) arises from damage to sensory neurons, manifesting in persistent pain. Progressive nerve degeneration can result in sensory loss and, in severe cases, amputation, exacerbating emotional distress and diminishing quality of life. Continuing investigation into DPN mechanisms is essential for advancing effective pain management. Our previous narrative review identified oxidative stress and central sensitization as key pathways linking hyperglycemia to nerve injury and pain. The present review provides an updated synthesis of evidence in this area. The original PubMed search term was expanded to include metabolic syndrome and pain modulation, and studies published between September 2021 and February 2026 were examined. The current findings reinforce earlier conclusions that alterations within the central nervous system and central sensitization are prominent features of painful DPN. Emerging evidence suggests that metabolic syndrome such as overweight may impair pain modulation in individuals with or without risk of diabetes. Future research should determine whether impaired pain modulation can serve as an early indicator of DPN, enabling earlier intervention. Clarifying these mechanisms may also help explain why medications that restore pain modulation show therapeutic promise for this complex chronic pain condition.

## Highlights

- This updated review reinforces that central nervous system alterations and central sensitization are key features of painful diabetic peripheral neuropathy.
- Changes in the central nervous system can diminish descending modulatory control over spinal sensory neurons, allowing enhanced pain signaling and thereby contributing to central sensitization.
- Metabolic syndrome such as overweight may further impair pain modulation in individuals with or without risk of diabetes, highlighting the need for further research.

## Graphical abstract

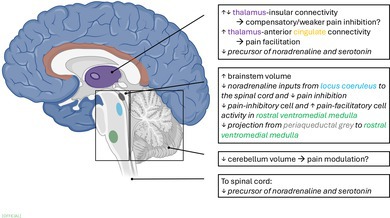

## 1 Introduction

More than 50% of people with diabetes develop diabetic peripheral neuropathy (DPN) [1]. In about one‐third of cases, nerve damage results in painful sensations such as burning, tingling, or shooting sensations in the feet. Some individuals may even experience discomfort from light touch such as putting on socks or brushing against bed sheets. Pain often worsens at night and disrupts sleep and ultimately reduces a person's ability to work or to carry out everyday activities. Over time, extensive nerve damage may result in sensory loss and unattended injury, increasing the risk of infection, ulcers, and ultimately amputation. Painful DPN exacerbates emotional distress and reduces quality of life. Although medications may help initially, they often require higher dosages and carry increasing side effects [1]. Therefore, understanding the mechanisms driving painful DPN is crucial for better pain management.

Our earlier review [2] highlighted oxidative stress and central sensitization as mechanisms linking hyperglycemia to nerve damage and pain in DPN. Hyperglycemia and reactive species directly activate sensory neurons to produce pain and indirectly enhance pain signaling via mitochondrial and inflammatory changes. Central nervous system alterations further weaken pain modulation, leading to central sensitization and increasing pain transmission.

The present review is to provide an updated synthesis on emerging evidence in this area. Given our prior findings that pain‐free adults at elevated risk of diabetes—reflected in high blood glucose and body fat mass—had weaker endogenous pain modulation [3, 4], the original PubMed search term was expanded to include obesity and multiple experimental pain modulation paradigms. The revised search string was: (Metabolic Syndrome OR Diabetes OR Hyperglycemia OR Obesity OR Adipose) AND (Pain) AND (Central Nervous System OR Central Sensitization OR “conditioned pain modulation” OR “offset analgesia” OR “temporal summation”). Terms in title case were mapped to Medical Subject Headings (MeSH). Daily PubMed alerts provided new search results to DY, who screened titles and abstracts and assessed full texts if relevant. From September 1, 2021 to February 28, 2026, PubMed sent 478 results (please see details in Supporting Information); 30 of which were included in the current review. Inclusion criteria were reviews/original research studies examining the central nervous system and pain in diabetes/overweight/obesity. Studies relevant to discussion were also included. Exclusion criteria were non‐English articles (*k* = 12), human pediatric research (*k* = 20), case reports, comments, letters, and preprints (together *k* = 43). Studies less relevant or unrelated to discussion were also excluded. Additional papers were identified through citation tracking and subscribed journal‐content alerts.

This review starts with a summary of animal and human research examining central nervous system involvement in painful DPN. It then presents new evidence supporting the presence of central sensitization in painful DPN. Lastly, it ends with emerging animal research on how hyperglycemia‐induced reactive species activate sensory neurons. No new evidence was identified for the other subsections of “Hyperglycemia and pain” covered in our previous review [2].

## 2 Central Nervous System in Painful Diabetic Peripheral Neuropathy

### 2.1 Animal Research

Unless otherwise noted, type 1 diabetes in animal studies was induced using a single injection of streptozotocin (in mice, 100–200 mg/kg; in rats, 35–65 mg/kg) [5]. Blood glucose levels of animal subjects and human participants are reported to illustrate a potential relationship between hyperglycemia and pain; however, definitive evidence establishing a direct link remains limited.

Four weeks after type 1 diabetes was induced in male rats (blood glucose = 30.0 mmol/L), animals showed increased heat and mechanical sensitivity, accompanied by a reduced level of tyrosine (precursor of noradrenaline) and tryptophan (precursor of serotonin) in the brain [6]. Similar reductions were also observed in the spinal cord of rats with type 2 diabetes (high‐fat diet + 30 mg/kg streptozotocin), where the lower spinal tryptophan levels were further associated with increased pain sensitivity [7].

Noradrenaline and serotonin play a part in descending pain modulation [8, 9, 10]. A deficiency in those neurotransmitters might increase the risk of central sensitization. For example, decreased noradrenaline inputs from the brainstem locus coeruleus to the spinal trigeminal nucleus weakened descending inhibition of orofacial pain in male mice with type 1 diabetes (blood glucose > 16.7 mmol/L), likely reflecting weaker activation of inhibitory trigeminal interneurons [11, 12]. It remains uncertain whether these neurochemical alterations stem directly from streptozotocin or from its damaging effects on pancreatic β‐cells and subsequent hyperglycemia. Given sex differences in pain processing [13], more research using female animals and models of type 2 diabetes is required to clarify how hyperglycemia affects brain amino acid metabolism and descending pain modulation.

In another brainstem region, the rostral ventromedial medulla, male mice exhibited increased activity of pain‐enhancing ON‐cells and a complete suppression of pain‐inhibiting OFF‐cells 3 weeks after type 1 diabetes induction (blood glucose > 13.9 mmol/L) [14]. Pharmacological activation of neurons in the midbrain ventrolateral periaqueductal gray, likely via metabotropic glutamate receptor 8, enhanced descending output to the rostral ventromedial medulla, restored the balance between ON‐ and OFF‐cells activity, and reduced pain sensitivity [14].

### 2.2 Human Research

Human research on central mechanisms in painful DPN has grown rapidly, with multiple recent reviews documenting structural, functional, and neurochemical alterations in the central nervous system [15, 16, 17]. Evidence presented here is selective and focuses on changes linked to weakened modulation of spinal sensory neurons and increased central sensitization.

#### 2.2.1 Thalamus–Insular Connectivity

In 10 participants with hyperalgesic‐type painful DPN (70% type 2 diabetes; 10% females; age 57 years; glycated hemoglobin [HbA1c] = 8.5% [69.1 mmol/mol]; 17 years with diabetes; 9 years with pain), a higher self‐reported pain score was associated with stronger thalamus and insular cortex connectivity on functional magnetic resonance imaging (MRI) while at rest [18]. This pattern was absent in the hypoalgesic type, despite similar demographic characteristics [18]. Given that ventromedial thalamic projections to the insula activate descending pain inhibition via the ventrolateral periaqueductal gray [19], the increased connectivity may reflect a compensatory engagement of pain‐inhibitory circuits during chronic pain.

In contrast, diffusion MRI tractography in 25 individuals with type 2 diabetes and painful neuropathy (40% females; age 60 years; HbA1c = 7.4% [57.0 mmol/mol]; 10 years with diabetes; 3 years with pain) revealed *reduced* connectivity between the ventrolateral‐posterior thalamic nuclei and the bilateral insular cortices [20]. This reduction was absent in the painless group (*n* = 13) with similar demographic and clinical profiles, suggesting a role of weaker thalamus–insular‐mediated pain inhibition in painful DPN.

The inconsistent findings between these two studies may reflect several factors: differences in imaging methods (functional MRI for cortical activity vs. tractography for white‐matter structure), variation in diabetes types, sex distribution, blood glucose levels, duration of diabetes and pain, or differences in the thalamic nuclei connecting to the insular cortex under different recording conditions. The theory of thalamus–insular‐mediated pain inhibition was based on rat models [19], which may partly explain the inconsistencies in human research.

Alternatively, reduced thalamic volume or diminished peripheral sensory input may contribute to weaker thalamic connectivity. In a cohort of 48 participants with long‐standing type 1 diabetes (21% females; age 50 years; diabetes duration 32 years; HbA1c = 8.2% [65.8 mmol/mol]), MRI indicated reduced thalamic gray matter volume in both painful and painless groups, although only 25% met the criteria for painful neuropathy, and they reported relatively mild symptoms [21]. The thalamic atrophy was associated with a loss of protective touch sensation in individuals with type 1 diabetes [21], consistent with the finding that reduced connectivity between the thalamus and insula in type 2 diabetes was associated with a lower intraepidermal nerve fiber density [20]. Together, these results suggest that weaker thalamus‐centered connectivity likely reflects structural thalamic volume loss and/or reduced afferent input from the periphery.

However, some complexities remain as structural MRI findings in 19 participants with type 1 diabetes and painful neuropathy (53% females; age 51 years; diabetes duration 30 years; HbA1c = 8.6% [70.0 mmol/mol]) showed that reduced thalamic volume was not associated with nerve degeneration [22]. Yet, in the same cohort, functional MRI demonstrated reduced thalamic‐somatosensory cortex connectivity, which was associated with peripheral nerve degeneration and higher pain intensity [23]. Despite these reductions in thalamic volume and connectivity, proton magnetic resonance spectroscopy revealed increased thalamic glutamate concentrations, which predicted painful neuropathy in this group [24]. A higher thalamic glutamate concentration is expected to enhance pain transmission, although it remains unclear how heightened thalamic neuroactivity contributes to pain alongside weaker connectivity with other brain regions.

#### 2.2.2 Thalamus–Cingulate Connectivity

In the diffusion MRI tractography study mentioned above [20], higher pain intensity was associated with a stronger connectivity between the ventrolateral‐posterior thalamic nuclei and the anterior cingulate cortex. The mediodorsal thalamic nuclei project to the anterior cingulate cortex, which, in turn, projects to the dorsolateral periaqueductal gray to facilitate pain [19]. Although different thalamic nuclei were reported across the studies, these findings collectively support a pain‐enhancing role of increased thalamus–cingulate connectivity.

Mechanistic animal research suggests that upregulated acid‐sensing ion channel 1a in glutamatergic neurons in the anterior cingulate cortex contributes to pain in diabetes [25]. This upregulation appears to result from activation of tumor necrosis factor‐α receptors on these glutamatergic neurons and subsequent engagement of nuclear factor‐κB pathways [25]. This new evidence suggests an additional central mechanism involving acid‐sensing ion channels, complementing the peripheral indirect pathway by which hyperglycemia promotes pain [2].

Of note, MRI showed a reduced cortical volume in the ventrobasal thalamus, insular, cingulate, primary somatosensory, and primary motor cortices in both painful (*n* = 77; 64% type 2 diabetes; 33% females; age 58 years; HbA1c = 8.7% [71.4 mmol/mol]; diabetes duration 18 years) and painless DPN groups (77 matched patients, except for having 24‐year diabetes duration) [26]. Within the painful group, individuals with the hypoalgesic type exhibited a greater reduction in the thalamus, primary somatosensory, and posterior cingulate cortices compared with those with the hyperalgesic type [26]. In contrast, the hyperalgesic type showed a greater reduction in the anterior cingulate cortex, which the authors suggested reflected neuronal loss caused by excessive neuronal activation [26]. This interpretation aligns with the pain‐enhancing role of thalamus–cingulate connectivity [19], suggesting that prolonged activation of cingulate‐mediated pain facilitation may contribute to anterior cingulate neuronal loss in the hyperalgesic type.

#### 2.2.3 Other Brain Regions

Reduced cortical volume was also observed in the cerebellum (Crus I, Crus II, and region VII) in 20 individuals with type 2 diabetes and painful DPN (60% females; age 61 years; HbA1c = 6.9%–9.1% [52–76 mmol/mol]; diabetes duration 18 years; pain duration 2 years) compared with 37 healthy controls [27]. This reduction was absent in the painless‐DPN group (*n* = 22), despite similar demographic characteristics [27]. The cerebellum contributes to sensorimotor, emotional, and cognitive dimensions of pain through its projections to forebrain areas [28]. Increased connectivity between the cerebellum and midbrain periaqueductal gray may enhance descending pain inhibition [28]. However, both increased and decreased cerebellar activities have been reported in neuropathic pain [28], indicating a complex role. Thus, further research is needed to elucidate the role of the cerebellum in painful DPN.

The painful‐DPN group also showed an increased brainstem volume [27]. The brainstem contains the locus coeruleus, which inhibits acute pain through descending projections to the spinal cord [9]. However, during chronic pain, its ascending projections to forebrain areas (e.g., prefrontal cortex, anterior cingulate cortex, and amygdala) may override the descending inhibition and result in pain facilitation [29, 30]. The brainstem also houses the rostral ventromedial medulla, where ON‐cells facilitate and OFF‐cells inhibit pain transmission, respectively [9]; disruptions in this balance amplify pain in diabetes [14, 31]. It remains unclear whether the increased brainstem volume reflects the locus coeruleus, the rostral ventromedial medulla, or both, and whether the enlarged volume reflects increased neural matter or initial pathological processes such as swelling. Further research is needed to identify the specific contribution of brainstem subregions in painful DPN.

### 2.3 Summary

**FIGURE 1:**
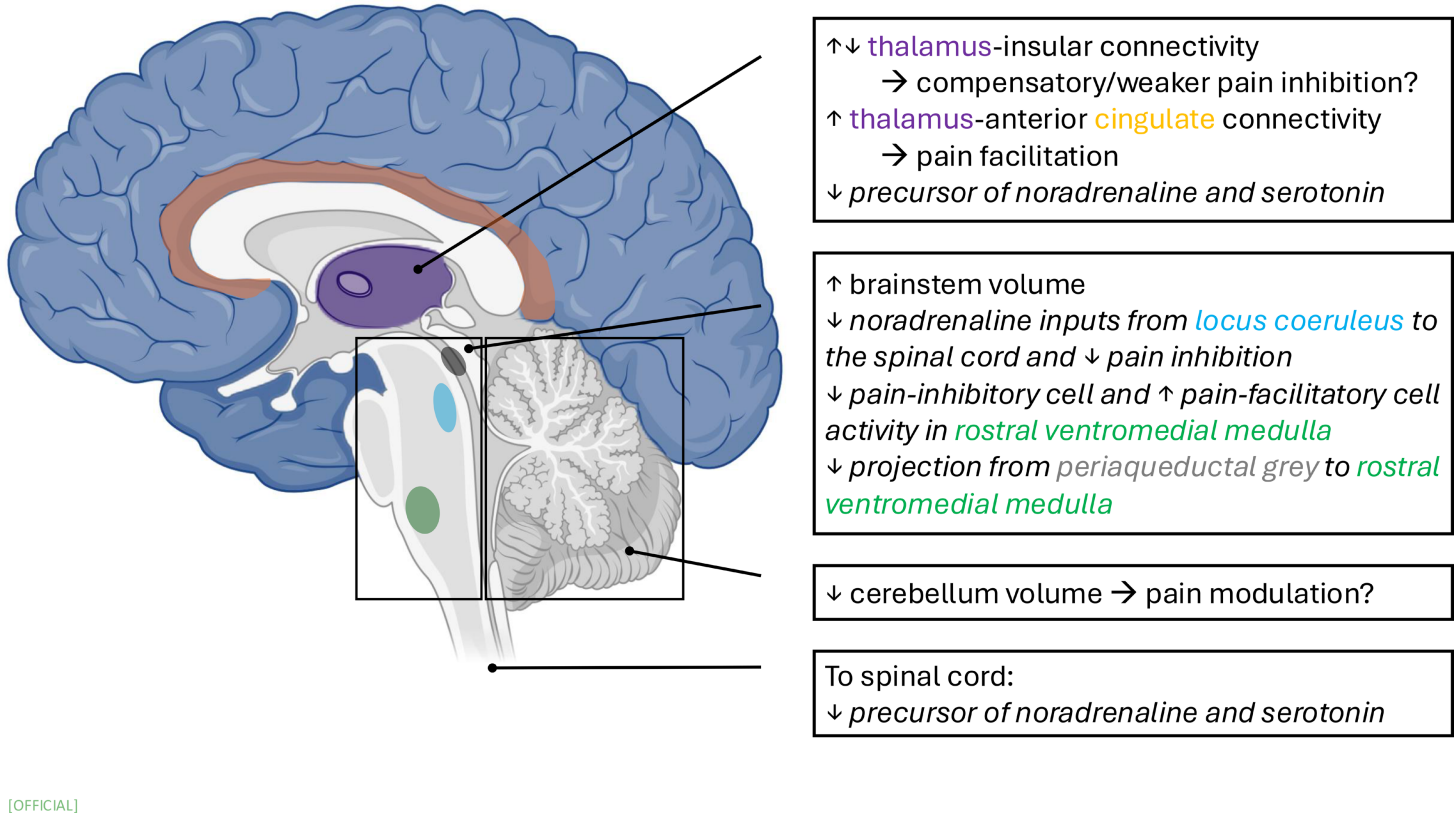
Alterations within the central nervous system in painful diabetic peripheral neuropathy. Collectively, the changes in forebrain–thalamus connectivity, midbrain–brainstem projections and activities, and hindbrain structures weaken descending modulation of spinal sensory neurons, thereby increasing central sensitization. Italics means animal research findings. Note that the insular cortex is hidden in this midsagittal plane. Brain icon was obtained from biorender.com.

Emerging evidence demonstrates central nervous system alterations in painful DPN that weaken descending pain modulation at the spinal level (Figure 1), thereby contributing to central sensitization. However, this must remain speculative as most studies were cross‐sectional rather than longitudinal, thus limiting causal inferences.

Of note, either increased or decreased thalamus–insular connectivity was observed with different imaging methods. The increased connectivity was found using functional MRI (indicating cortical gray‐matter activity), whereas the decreased connectivity was found using diffusion MRI tractography (indicating white‐matter structure). Whether persistent pain, due to compromised connections between thalamic and insular neurons, triggered activity in these neurons in a positive loop deserves further investigation. Therefore, replicating similar neuroimaging results is essential to understand the role of thalamus–insular connectivity in painful DPN and whether patient characteristics such as duration of diabetes and pain change the strength of the connectivity. For prospective researchers, methodological limitations and future directions for MRI research in DPN have been discussed elsewhere [32]. Lastly, the increased thalamus–insular connectivity was found in the hyperalgesic but not the hypoalgesic group [18], emphasizing necessary subgroup comparisons in future research.

## 3 Central Sensitization in Painful Diabetic Peripheral Neuropathy

Central sensitization refers to an increased responsiveness of spinal sensory neurons, resulting in amplified pain signaling [33]. Key mechanisms include overactive glutamatergic excitation, reduced γ‐aminobutyric acid (GABA) inhibition, and glial cell‐mediated neuroinflammation. Recent advances have primarily emerged in the latter two areas, with most evidence derived from animal studies.

### 3.1 Glutamatergic Over‐Transmission

Activation of the spinal glutamatergic NR2A subunit increased reactive species production in mice with type 1 (streptozotocin) or type 2 diabetes (db/db model with genetic deficiency in the leptin receptor) [5] with neuropathic pain (blood glucose > 16.7 mmol/L) [34]. Specifically, NR2A activation enhanced proinflammatory signaling via co‐expressing toll‐like receptor 2 and subsequent nuclear factor‐κB activation [34], leading to mitochondrial dysfunction and reactive‐species overproduction [34]. Elevated spinal reactive species further amplified inflammation through the NR2B glutamate receptors and the NOD‐like receptor family pyrin domain‐containing 3 (NLRP3) in male rats with type 2 diabetes (high‐fat and high‐sugar diet + 35 mg/kg streptozotocin; blood glucose = 30.0 mmol/L) [35]. Spinal NR2B receptors could also have been upregulated when advanced glycation end‐products—proinflammatory molecules generated under hyperglycemic conditions—bound to their receptors, resulting in hyperalgesia in male mice with type 1 diabetes (blood glucose > 16.7 mmol/L) [36]. Reducing reactive species or NR2B upregulation alleviated pain [35, 36]. Together, these findings suggest that interactions between glutamatergic NR2A and NR2B subunits may sustain glutamatergic transmission and central sensitization in diabetes.

### 3.2 GABA Disinhibition

Animal research suggests that postsynaptic (central) GABA inhibition is mediated by both GABA_A_ and GABA_B_ receptors [37]. Four weeks after the onset of type 1 diabetes in female rats (blood glucose > 20.0 mmol/L), dysfunction of the potassium‐chloride co‐transporter‐2 resulted in GABA_A_‐mediated disinhibition [37]. Concurrent blockade of spinal GABA_B_ receptors aggravated this disinhibition, suggesting that GABA_B_ receptors remained functional and were compensating for GABAergic inhibition [37]. Although GABA_B_ remained functioning at 8 weeks of diabetes, its compensatory effect was insufficient to counter the dysfunctional GABA_A_ [37]. For a comprehensive review of GABA‐mediated spinal inhibition and methodological approaches to assessing spinal disinhibition, readers are directed to a recent review [38].

GABA‐mediated spinal inhibition has been used to distinguish painful from painless DPN [38]. In one study, individuals with painful DPN (*n* = 42; 74% type 2 diabetes; 43% females; age 62 years; HbA1c = 7.0% [53.0 mmol/mol]; diabetes duration 13 years) showed reduced spinal inhibition, whereas those with painless DPN (*n* = 62) showed increased spinal inhibition, despite similar demographic characteristics and neuropathy severity [39]. The authors attributed the increased inhibition in the painless group to compensatory activity of intact GABA_B_ receptors [39]. However, it is unclear why GABA_B_ remained functioning in the painless group.

Peripherally, spinal disinhibition in the painful group was associated with greater burning pain and a pattern of combined mechanical/heat detection hyposensitivity alongside pain hypersensitivity (i.e., slower to detect but quicker to report pain) [40]. Centrally, spinal disinhibition would imply impaired pain modulation; however, the painful and painless groups did not differ in conditioned pain modulation measured in the upper extremities [40]. A caveat is that individuals with DPN tend to exhibit sensory alterations in their extremities that could confound the results of conditioned pain modulation [41]. Despite this, the authors [39, 40] and others [38] suggested that individuals with spinal disinhibition and pain hypersensitivity may benefit from spinally acting medications that restore pain modulation, such as duloxetine and gabapentin.

The efficacy of gabapentin in restoring spinal inhibition was examined in 71 individuals with type 2 diabetes and painful DPN (72% females; age 49 years; blood glucose = 7.2 mmol/L; HbA1c = 9.4% [79.0 mmol/mol]; 11 years with diabetes; 8 years with pain; 5/10 pain intensity at baseline) [42]. Greater spinal disinhibition at baseline predicted a better treatment response, with pain reduced to 3/10 after 2 weeks of gabapentin, independent of changes in blood glucose levels [42]. The analgesic effect of gabapentin was also associated with its ability to restore spinal GABA inhibition [42].

Although gabapentin was designed as a lipophilic GABA analogue, it does not have a high affinity for either GABA_A_ or GABA_B_ receptors [43]. Therefore, the mechanisms through which it restores spinal inhibition require further clarification. Moreover, because blood glucose levels did not differ between individuals with spinal inhibition versus disinhibition [39] or before/after gabapentin treatment [42], future studies should explore whether other metabolic disturbances such as overweight or hypertension are linked to pain [44]. Supporting this possibility, individuals with overweight/obesity (*n* = 13; no known clinical conditions) showed weaker spinal inhibition compared with normal‐weight individuals (*n* = 12), although many in the overweight/obesity group had prediabetes (8/13; HbA1c > 6.0% [42.1 mmol/mol]) [45].

### 3.3 Overactive Glial Cells

Recent reviews [17, 46, 47] highlight neuroinflammation as a mechanism for painful DPN. Specifically, spinal microglia and astrocytes release proinflammatory cytokines—including interleukin‐1β, interleukin‐6, tumor necrosis factor‐α, and brain‐derived neurotrophic factor (BDNF)—which activate extracellular signal‐regulated kinases in peripheral sensory neuron terminals [46]. These kinases interact with Nav1.7 and Nav1.8 sodium channels to increase presynaptic glutamate release [46]. The resulting enhanced postsynaptic activation of glutamate receptors in central sensory neurons may facilitate nociceptive neurotransmission and promote central sensitization. Targeting microglia and astrocytes through pharmacological agents such as minocycline or other nonpharmacological interventions such as electroacupuncture shows promise for alleviating painful DPN [47].

#### 3.3.1 Microglia

Minocycline, a drug that inhibits microglial activation, reduced mechanical allodynia (pain evoked by touch) in male rats with type 1 diabetes for 3 weeks (blood glucose > 15.0 mmol/L) [48]. The analgesic effect was attributed to minocycline's inhibition of the NR2B subunit of spinal glutamate receptors, thereby attenuating excessive spinal pain transmission [48]. These researchers also reported decreased expression of spinal NR2B glutamate receptors in rats with painless diabetic neuropathy (< 15% hyperalgesia from the baseline value) [49]. Given the proinflammatory and pain‐enhancing effects of the NR2B subunit [35], this receptor subunit may represent a promising future therapeutic target.

Minocycline may also relieve pain by reducing microglial production of chemokine CXCL12 that binds to CXCR4 receptors in the anterior cingulate cortex to sensitize glutamate receptors (shown in male mice with type 1 diabetes; blood glucose > 20.0 mmol/L) [50]. Alleviating pain by reducing anterior cingulate neuronal activity is consistent with the proposed pain‐facilitating effect of thalamus–cingulate connectivity [19].

In a study [44], individuals with type 2 diabetes and painful DPN (*n* = 78; 49% females; age 59 years; HbA1c = 9.0% [74.9 mmol/mol]; 10 years with diabetes; 5 years with pain) had higher serum BDNF levels than those with painless DPN (*n* = 65; with similar demographic characteristics). The painful DPN group also had a higher body mass index (28.1 vs. 27.0 kg/m^2^) and diastolic blood pressure (81.0 vs. 79.9 mmHg). Together, elevated serum BDNF, higher body mass index, and higher diastolic blood pressure predicted both the presence of painful DPN and greater self‐reported pain [44]. The absence of group differences in blood glucose levels suggests that overweight and hypertension in diabetes may contribute to painful DPN independently of hyperglycemia. A pain‐overweight link irrespective of glycemic control was also observed in a prospective cohort [51]. However, the clinical significance of these relatively small increases in body weight and blood pressure requires further research.

Regarding potential mechanisms, in overweight and obesity, expanded fat cells secrete proinflammatory cytokines such as interleukin‐6 and tumor necrosis factor‐α [52]. Although fat cells also secrete anti‐inflammatory adiponectin [52, 53], paradoxically, adiponectin mediated the association between free fatty acids and proinflammatory tumor necrosis factor‐α in people with type 2 diabetes [54]. Consequently, chronic inflammation due to overweight/obesity may enhance the risk of chronic pain in diabetes. Other mechanisms linking pain with obesity, especially excess visceral fat, have been discussed in a recent review [53]. That said, in a large cross‐sectional study (*N* = 7090), obesity predicted DPN but not painful DPN [55], presumably mediated by obesity‐related hyperinsulinemia [56]. Thus, overweight/obesity is unlikely to be the sole contributor for painful DPN. Other interacting risk factors require further investigation.

#### 3.3.2 Astrocytes

Astrocytes activate more slowly than microglia. In male rats, mechanical hypersensitivity emerged 1 week after type 1 diabetes induction (blood glucose > 28.0 mmol/L), whereas astrocytic activation in the midbrain ventrolateral periaqueductal gray appeared after another 2 weeks [57]. Inhibiting astrocytic activation with a designer drug alleviated both pain and related anxiety in rats with painful DPN, while activating astrocytes in control rats (blood glucose < 8.0 mmol/L) induced mechanical allodynia [57]. These researchers also reported similar effects of astrocytic activation in the basolateral amygdala [58], the paraventricular thalamic nucleus [59], and the rostral ventromedial medulla [60].

Because astrocytic activation can induce pain even under normal glucose levels, underlying mechanisms require further research. One potential mechanism may involve local inhibitory circuits. In the paraventricular thalamic nucleus, activation of GABAergic neurons reduced pain, whereas inhibition of these neurons increased pain [59]. The authors proposed that astrocytic activation may disrupt this GABAergic inhibitory control, resulting in dysregulated descending pain modulation [59].

Another mechanism involved the proinflammatory effect of astrocytic activation. In male rats with type 1 diabetes (blood glucose = 27.0 mmol/L; HbA1c = 7.0% [53.0 mmol/mol]), a 4‐week treatment with semaglutide (a glucagon‐like peptide 1 receptor agonist) reduced plasma levels of glucose (to 19.0 mmol/L; HbA1c to 5.5% [36.6 mmol/mol]) and advanced glycation end‐products. Semaglutide also alleviated mechanical allodynia and thermal hypersensitivity [61], likely through its inhibitory effects on spinal astrocytes and microglia, which subsequently reduced levels of proinflammatory cytokines (interleukin‐1β, interleukin‐6, tumor necrosis factor‐α) and oxidative stress [61]. Notably, however, in the hippocampus of male rats with type 1 diabetes (blood glucose > 16.7 mmol/L) and mechanical hypersensitivity, microglial activity increased, whereas astrocytic activity decreased [62]. These contrasting patterns suggest that astrocytic contributions to pain regulation may vary across brain regions and thus require further research.

### 3.4 Summary

Emerging evidence supports the presence of central sensitization in painful DPN. This includes heightened excitatory transmission driven by the interaction between NR2A and NR2B glutamate receptors, reduced inhibitory controls linked to impaired GABA_B_ receptor signaling, and glial cell‐mediated neuroinflammation supported by a growing body of research on astrocytes. Note, however, results are mainly based on animal studies and should be interpreted with caution.

## 4 Direct Pain Activation by Hyperglycemia‐Induced Reactive Species

Hyperglycemia‐induced reactive species can directly contribute to pain by activating ion channels expressed on pain receptors [2]. New evidence strengthens this link and identifies an additional contributor, the transient receptor potential melastatin 7 channel. Antioxidant treatments show promise for mitigating these effects. However, these findings are also derived primarily from animal research, and their applicability to humans remains to be established.

### 4.1 Transient Receptor Potential Vanilloid 1 (TRPV1)

In a study included in our previous review, a 1‐week treatment of antioxidant α‐lipoic acid (60 and 120 mg/kg) downregulated TRPV1 channels and reduced pain in female rats with type 1 diabetes (blood glucose > 15.0 mmol/L) [63]. A similar analgesic effect was observed in a more recent study using a 2‐week treatment of α‐lipoic acid (50 mg/kg) [64]. However, both studies used female rat models of type 1 diabetes. Future research in male animals and in models of type 2 diabetes is needed to evaluate the generalizability of α‐lipoic acid's pain‐relieving effects.

In male rats with type 1 diabetes (blood glucose > 16.7 mmol/L), a 4‐week treatment with antioxidant Ajugarin‐I (a diterpene extracted from Ajuga bracteosa leaves) reduced pain by suppressing oxidative stress, lowering inflammatory cytokine production, and downregulating spinal TRPV1 channel expression [65]. Because Ajugarin‐I also improved pancreatic function and reduced hyperglycemia [65], future studies could investigate whether improvements in glycemic control contribute to the observed reduction in TRPV1 channel expression.

### 4.2 Transient Receptor Potential Ankyrin 1 (TRPA1)

In male mice with type 2 diabetes (db/db; blood glucose > 27.8 mmol/L; HbA1c > 8.0% [63.9 mmol/mol]), spinal injection of methylglyoxal increased both heat and mechanical pain [66]. This heightened pain was mediated by methylglyoxal‐induced elevation in calcium responses (indicative of neuroexcitation) in spinal sensory neurons involved in pain transmission [66]. Importantly, removal or inhibition of spinal TRPA1 channels interrupted the cascade responsible for initiating and sustaining methylglyoxal‐induced pain hypersensitivity [66].

Methylglyoxal is a by‐product of glucose metabolism and increases during periods of hyperglycemia [67]. Therefore, the above findings link hyperglycemia with enhanced nociceptive neurotransmission. Notably, by using the db/db genetically mutated model of type 2 diabetes, the authors [66] avoided the confounding pain‐enhancing effects of streptozotocin through TRPA1 channels [68].

### 4.3 Transient Receptor Potential Melastatin 7 (TRPM7)

In male mice with type 1 diabetes (blood glucose > 15.3 mmol/L), the levels of mitochondrial and cytosolic reactive species (oxidants) increased while the levels of antioxidants decreased [69]. These reactive species activated TRPM7 channels, which increased calcium influx to the mitochondria [69]. Excess calcium disrupts the function of the mitochondrial inner membrane where the respiratory chain is located, impairing ATP production and further amplifying reactive species production [70], which, in turn, reactivates TRPM7 channels [69]. Administration of antioxidants (selenium and curcumin) inhibited TRPM7 activity, reduced neuropathic pain, and improved blood glucose levels in these mice [69]. Future research could confirm these findings using animal models of type 2 diabetes or examine whether streptozotocin influences TRPM7 channel activity.

## 5 Limitations

Unlike a regular systematic review, the first author continuously screened articles from PubMed alerts, journal‐content alerts, and citation tracking. It is thus hard to generate a flow diagram retrospectively to indicate article selection. As PubMed was the only database being used, articles listed exclusively in other databases could have been missed. Article selection might also have been biased given the sole reviewer. For transparency, all search results in PubMed are provided as Supporting Information for public access.

For the included papers, most human studies on the central nervous system had cross‐sectional rather than longitudinal designs. It remains unclear whether alterations in the central nervous system develop before or after painful DPN. In addition, studies on the link between central sensitization and pain‐hyperglycemia were mostly carried out in animal models. Applicability to humans remains to be established.

Lastly, sample characteristics and research methods varied across studies (see details in Supporting Information), which could have contributed to inconsistent findings in human neuroimaging studies. As literature grows in the future, collecting study statistics for quantitative analysis of study heterogeneity and effect size would be worthwhile.

## 6 Conclusions and Future Directions

Consistent with conclusions from our previous review [2], emerging evidence continues to support alterations in the central nervous system and the presence of central sensitization in painful DPN. These central changes likely weaken descending modulation of spinal sensory neurons (Figure 1), thereby allowing enhanced pain signaling and contributing to central sensitization. However, more research is needed to clarify the role of descending pain modulation in painful DPN.

Pain modulation can be assessed using the conditioned pain modulation paradigm, which measures the body's capacity to inhibit weaker pain during exposure to stronger pain elsewhere [10]. Some studies suggest that conditioned pain modulation is impaired in individuals with painful DPN [71, 72, 73]. However, the painful and painless groups did not differ in conditioned pain modulation tested in the upper extremities [40], an area where sensory alterations in DPN might have confounded results [41]. To overcome this limitation, we applied an ice block to the temple as the stronger pain stimulation and assessed its modulation on weaker pressure and heat pain stimulation in the upper limb [4]. Using this paradigm, we observed impaired conditioned pain modulation in pain‐free adults with prediabetes (*n* = 10; 5.7 ≤ HbA1c < 6.5%) and type 2 diabetes (*n* = 3; ≥ 6.5%) [4].

Findings from the current review also suggest pain‐enhancing effects of excess weight in people with diabetes. This aligns with our previous findings, which showed that pain‐free adults at risk of diabetes, characterized by high blood glucose and high body fat mass measured via dual‐energy X‐ray absorptiometry, demonstrated weaker conditioned pain modulation compared with adults without these risk factors [3, 4]. These observations suggest that deficits in conditioned pain modulation may develop early in the trajectory of painful DPN, highlighting its potential as an early warning marker. Nonetheless, the neurophysiological mechanisms underlying this dysfunctional pain modulation remain unknown.

The primary mechanism underlying conditioned pain modulation involves a descending pathway from the locus coeruleus in the brainstem to the spinal cord, where noradrenaline release activates α_2_‐adrenoceptors [9]. This mechanism may explain the clinical effectiveness of duloxetine which restores noradrenaline levels and thereby enhances central pain inhibition in the treatment of painful DPN [1]. Unexpectedly, brainstem volume was greater in individuals with painful DPN than in healthy controls [27]. Although this could suggest a link with impaired pain modulation in DPN, the finding remains difficult to interpret. It is unknown which specific brainstem regions contribute to the enlargement, whether the increased volume reflects greater neuronal density or initial pathological processes such as swelling, and whether it has any connection to conditioned pain modulation. Given these uncertainties, examining the role of locus coeruleus in painful DPN, such as through neuromelanin‐based magnetic resonance imaging [74], may provide valuable insights.

Together, findings from this updated narrative review reinforce previous conclusions that painful DPN involves alterations within the central nervous system and the presence of central sensitization. New findings suggest that metabolic syndrome may exert additional disruptive effects on pain modulation in individuals with or without diabetes risk. To conclude, the agenda for future research includes:

1. Clarify whether impaired pain modulation serves as an early warning sign of DPN.
2. Elucidate mechanisms that restore descending pain inhibition in painful DPN.
3. Use randomized‐controlled trials or longitudinal designs to evaluate the causal role of glycemic control, weight loss, and alterations of the central nervous system in painful DPN.

## Author Contributions

All authors have contributed significantly and in keeping with the latest guidelines of the International Committee of Medical Journal Editors. Each author's contribution to the manuscript is described as follows: **Di Ye:** conceptualization, methodology, validation, investigation, data curation, writing – original draft, review and editing, visualization. **Timothy J. Fairchild**, **Lechi Vo**, and **Peter D. Drummond:** writing – review and editing, mentorship.

## Funding

The authors have nothing to report.

## Ethics Statement

The authors have nothing to report.

## Consent

The authors have nothing to report.

## Conflicts of Interest

The authors declare no conflicts of interest.

## Supporting information


**Table S1:** jdb70223‐sup‐0001‐Supinfo.xlsx.

## Data Availability

The data that supports the findings of this study are available in the Supporting Information of this article.
